# Severe food insecurity associated with mortality among lower-income Canadian adults approaching eligibility for public pensions: a population cohort study

**DOI:** 10.1186/s12889-020-09547-y

**Published:** 2020-10-01

**Authors:** Fei Men, Valerie Tarasuk

**Affiliations:** Department of Nutritional Sciences, University of Toronto, Medical Sciences Building, Room 5366, 1 King’s College Circle, Toronto, ON M5S 1A8 Canada

**Keywords:** Food insecurity, Mortality, Health, Pension, Seniors, Canada

## Abstract

**Background:**

The prevalence of food insecurity among adults over 65 in Canada is less than half of that among adults approaching 65, possibly due in part to the public pension universally disbursed from the age of 65. Given research associating food insecurity with higher risk of premature mortality, our objective was to determine the likelihood that food-insecure adults with incomes below the national median would live past 65 to collect the public pension.

**Methods:**

We linked respondents of the Canadian Community Health Survey 2005–15 to the death records from the Canadian Vital Statistics Database 2005–17. We assessed household food insecurity status through a validated 18-item questionnaire for 50,780 adults aged 52–64 at interview and with household income below the national median. We traced their vital status up to the age of 65. We fitted Cox proportional hazard models to compare hazard of all-cause mortality before 65 by food insecurity status while adjusting for individual demographic attributes, baseline health, and household socioeconomic characteristics. We also stratified the sample by income and analyzed the subsamples with income above and below the Low Income Measure separately.

**Results:**

Marginal, moderate, and severe food insecurity were experienced by 4.1, 7.3, and 4.5% of the sampled adults, respectively. The crude mortality rate was 49 per 10,000 person-years for food-secure adults and 86, 98, and 150 per 10,000 person-years for their marginally, moderately, and severely food-insecure counterparts, respectively. For the full sample and low-income subsample, respectively, severe food insecurity was associated with 1.24 (95% CI: 1.06, 1.45) and 1.28 (95% CI: 1.07, 1.52) times higher hazard of dying before 65 relative to food security. No association was found between food insecurity and mortality in the higher-income subsample.

**Conclusions:**

Severely food-insecure adults approaching retirement age were more likely to die before collecting public pensions that might attenuate their food insecurity. Policymakers need to acknowledge the challenges to food security and health faced by working-age adults and provide them with adequate assistance to ensure healthy ageing into retirement.

## Background

Household food insecurity, inadequate or uncertain access to food due to financial constraints, is a serious public health problem in many high-income countries. In Canada in 2017–18, one in eight households reported some experience of food insecurity [1]. Vulnerability to household food insecurity is tightly linked to other markers of social and economic disadvantage. Risk in Canada is highest among households characterized by low income; absence of a university education; reliance on social assistance, Employment Insurance or Workers’ Compensation; Indigenous identity; renting rather than owning one’s home; and single-parent female-led households or unattached individuals [2]. The lowest risk of food insecurity is found among households reliant on seniors’ pensions or other retirement income sources [1, 2]. Consistent with this finding, there is a much lower prevalence of household food insecurity among Canadians over 65 years of age compared to younger adults. In 2011–12, 2.5% of adults 65 years and older lived in moderately or severely food-insecure households, compared to 7.1% for 45–64 year-olds, 9.4% for 34–44 year-olds, and 10.4% for 20–34 year-olds [3]. Similarly, seniors in the US appear less impacted by food insecurity than other groups [4]. In both countries, the lower rates of food insecurity among seniors have been attributed to the protective effect of pension programs [5, 6].

Old Age Security (OAS) and Guaranteed Income Supplement (GIS) constitute the backbone of the public pension system in Canada. All Canadians aged 65 and above are entitled to OAS if they have lived in Canada for ten years or more after the age of 18 [7]. This public pension program was implemented to help low-to-moderate income residents avoid poverty after retirement, and it is considerably more generous than other income assistance programs in Canada. The amount of OAS received depends on the length of time residing in Canada as an adult and one’s income, subject to clawback for people with net incomes above an inflation-adjusted threshold ($79,054 in 2020) [7, 8]. In addition, low-income OAS recipients may receive the means-tested GIS [9]. For a single person with no other income sources, the maximum combined OAS-GIS annual income is roughly double the amount of social assistance income available to someone on welfare in Canada [10, 11]. Recent analyses of food insecurity prevalence rates among low-income unattached adults aged 55 to 74 suggest that reaching 65, the age of entitlement for OAS and GIS, is associated with a 50% reduction in the prevalence of moderate or severe food insecurity and better mental and functional health [5, 12]. The authors ascribed the outcomes to the greater adequacy and security of income enjoyed by those 65 years and older.

Food insecurity has been linked to poorer health [13–19], poorer disease management [20–23], and higher risk of premature mortality [24–28]. We recently found that marginal, moderate, and severe food insecurity were respectively associated with a 10, 11, and 37% higher risk of dying before 83 among Canadian adults 18 years and older [24]. Severe food insecurity was associated with all causes of death except cancer, with particularly strong linkage to deaths from infectious-parasitic diseases, unintentional injuries and suicides [24]. While we adjusted for age at interview in the models of our study, we did not interrogate the relationship between food insecurity and mortality across ages. Of particular interest are non-senior adults approaching retirement age, who are more likely to not only experience food insecurity but also to report poor health compared to their recently retired counterparts at or above 65 [5, 12]. Given the literature on household food insecurity and adverse health outcomes among near-retirement adults, we hypothesized that food-insecure adults from lower-income households who are nearing retirement age are more likely to die before becoming eligible to collect a public pension at the age of 65 compared to their food-secure counterparts. To test this hypothesis, we linked administrative death records to national health survey data to examine the association between household food insecurity status and pre-65 mortality among Canadian adults. Therefore, we restricted this analysis to adults with incomes below the population median, among whom food insecurity is most prevalent and most likely to be alleviated by receipt of public pension [1, 5]. We further explored the relationship between food insecurity status and mortality among those with low incomes, recognizing that inadequate income is a major barrier to both food access and health management [1, 29].

## Methods

### Study population

We linked the cycles of Canadian Community Health Survey (CCHS) spanning 2005–15 to the Canadian Vital Statistics Database (CVSD) 2005–17.

CCHS is an annual cross-sectional survey, with each cohort representing 98% of the Canadian non-institutionalized population aged 12 and above. The survey data comprise roughly 130,000 respondents per two-year cycle. CCHS contains a module assessing household food insecurity; however, provincial/territorial participation in the module was optional in certain cycles (Supplementary table).

CVSD is an administrative database containing the date and cause of deaths registered by all Canadian jurisdictions. All individuals aged 12 or older who died between January 1, 2000 and December 31, 2017 are linkable to CCHS, with minimal mislinkage [30].

After linking CCHS to CVSD, we excluded respondents with invalid or missing food insecurity data, age at interview over 64, or household income above population median (Supplementary figure). We also excluded respondents under age 52 at interview to focus on adults approaching retirement age and minimize right-censoring since 52 is the youngest age of the sampled interviewees with vital status traceable to age 65 by 2017. With interview being the onset of vital status tracing, we built an analytical sample of 354,000 person-years from 50,780 adults aged 52 to 64 at the time of their CCHS interview in 2005–15 for survival analysis. All sampled individuals were observed only once, at the interview. Depending on the year and age of interview, the respondents’ vital status was traced for 2–12 years after the interview up to the end of 2017. Following Statistics Canada’s reporting rules, numbers of observations shown in this paper were rounded to the nearest digit of five for identity protection. This study was approved by the Health Sciences Research Ethics Board at University of Toronto.

### Measurements

The outcome was time elapsed since age at interview until observed death before age 65 by December 31, 2017. Individuals with no CVSD records were assumed alive. Those alive and aged 64 or younger on the last day of 2017 (i.e. with unobservable vital status on their 65th birthday) were right-censored.

Household food insecurity over the prior 12 months was measured using the Household Food Security Survey Module, a validated 18-item scale of severity developed by the US Department of Agriculture and adapted by Health Canada [31]. The measure enables identification of food-secure, marginally food-insecure, moderately food-insecure, and severely food-insecure households based on levels of food deprivation ranging from worrying about food running out to going hungry without eating for days (Table 1).

**Table 1:** Food insecurity level, based on CCHS 18-item questionnaire

Status	Measurement	Interpretation
Food-secure	Affirmed no item on either the 10-item adult food security scale or 8-item child food security scale	No report of income-related problems of food access.
Marginally food-insecure	Affirmed no more than 1 item on either scale	Some indication of worry or an income-related barrier to adequate, secure food access.
Moderately food-insecure	Affirmed 2 to 5 items on the adult scale or 2 to 4 items on the child scale	Compromise in quality and/or quantity of food consumed by adults and/or children due to a lack of money for food.
Severely food-insecure	Affirmed more than 5 items on the adult scale or more than 4 items on the child scale	Disrupted eating patterns and reduced food intake among adults and/or children due to a lack of money for food.

Consistent with prior literature [24, 25], we controlled for covariates that may confound the association between food insecurity and pre-65 mortality, including individual demographic characteristics, baseline health, and household socioeconomic characteristics. Demographic covariates included respondent’s sex (male/female) and age at interview (integer years 52–64). Health covariates included smoking status (never/former/current smoker), alcohol consumption in the previous year (none/once a year to once a week/more than once a week), and self-reported number of chronic conditions among cancer, hypertension, effects of stroke, diabetes, and heart diseases (none/1/2/3 or more). We further controlled for socioeconomic characteristics including the highest level of educational attainment in the household (high school incomplete/high school graduate/some college/college degree), respondent’s Indigenous status (non-Indigenous/Indigenous), homeownership (renter/homeowner), household type (couples with children/couples without children/single parents/individuals and other types), and before-tax household income relative to the Low Income Measure (LIM) (below LIM/at or above LIM). With cut-offs set to half the national income median, LIM is a measure of relative income poverty adjusted by household size. Missing values for covariates were labelled separately from the non-missing categories and kept in the analysis.

### Analyses

We first described our sample, contrasting the means of the predicting variables for those who died before age 65 and those who didn’t. T-tests were employed to determine statistical significance of the between-group differences; sample weights from the CCHS survey were applied to correct sampling bias. Next, we computed the average crude mortality rate for individuals from each food insecurity level, dividing the number of deaths by length of follow-up (i.e. total number of person-years traced). We also calculated the mean age at death by food insecurity level for individuals with observed death between ages 52 and 64.

We used Cox proportional hazard models to estimate the hazard ratio (HR) of pre-65 mortality by food insecurity level adjusting for confounders. We employed the Schoenfeld test to verify whether our model met the proportional hazard assumption. Two variables, age at interview and number of chronic conditions, were found to be in violation of the assumption. We followed the standard approach and stratified the two variables such that each stratum assumed a different baseline hazard function while the coefficients of the other covariates remained constant across strata [32, 33]. With the Schoenfeld test passed (global test *p* > 0.1), we proceeded with the univariate analysis associating food insecurity with mortality; we then added the demographic and health covariates, confounders with presumably direct impact on one’s lifespan; we finally added the socioeconomic characteristics, the distal determinants of mortality. We also examined the subsamples below and above LIM, recognizing the greater prevalence and severity of food insecurity and potentially elevated risk of health problems among adults with incomes below LIM, and their greater potential to benefit from the receipt of public pensions should they reach 65. We reasoned that the food insecurity among the below-LIM subsample would be more responsive to the receipt of public pensions than that of the above-LIM subsample given the former’s lower pre-retirement level of financial resources. Analyses were done using Stata 15.1. *P* < 0.05 was considered significant.

## Results

**Fig. 1:**
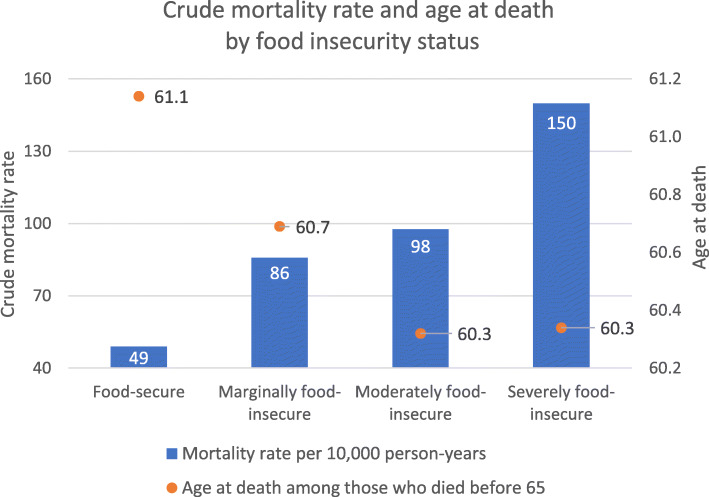
Crude mortality rate and age at death by food insecurity status. Crude mortality rate per 10,000 person-years (blue bars; *N* = 354,000 person-years) was obtained by dividing observed number of pre-65 deaths by number of person-years traced for each food insecurity level in the full sample. Mean age at death (orange dots; *N* = 2075 persons) was computed for each food insecurity level by taking the average age at death among those with observed deaths before 65 by 2017

Marginal, moderate, and severe household food insecurity affected 4.1, 7.3, and 4.5% of the sample, respectively (Table 2). A total of 2075 adults died between ages 52 and 64. Compared to adults with observed deaths before 65, those who were alive at age 65 or censored before age 65 were more likely to be food-secure (84.7% versus 68.2%), healthier, and socioeconomically better off (Table 2). The crude mortality rate for food-secure adults was 49 per 10,000 person-years (1460 deaths); the comparable figures for marginally, moderately, and severely food-insecure adults were 86 (125 deaths), 98 (255 deaths), and 150 (235 deaths) per 10,000 person-years, respectively (Fig. 1). Among those who died before 65, moderately and severely food-insecure adults died on average 0.8 year (9.6 months) earlier than their food-secure counterparts (*p* < 0.05) (Fig. 1).

**Table 2:** Sample characteristics by vital status at age 65

	Alive/censored by 65		Died before 65		Total	
Respondents N	48,705		2075		50,780	
Weighted population N	21,239,400		730,500		21,969,800	
Covariates	Mean	SD	Mean	SD	Mean	SD
Household food insecurity status						
Food-secure	84.7	36.0	68.2	46.6	84.2	36.5
Marginally food-insecure	4.0	19.6	7.0	25.4	4.1	19.8
Moderately food-insecure	7.0	25.5	14.7	35.4	7.3	25.9
Severely food-insecure	4.3	20.2	10.3	30.3	4.5	20.6
Demographic						
Female	54.1	49.8	40.1	49.0	53.7	49.9
Age at interview (years)	58.2	3.8	57.2	3.2	58.1	3.8
Health						
Number of chronic conditions						
No chronic condition	58.5	49.3	40.1	49.0	57.8	49.4
1 condition	28.0	44.9	32.2	46.7	28.2	45.0
2 conditions	10.4	30.6	17.9	38.4	10.7	30.9
3+ conditions	2.5	15.5	8.1	27.3	2.7	16.1
Missing	0.6	7.9	1.5	12.4	0.7	8.1
Smoker status						
Never smoked	29.5	45.6	16.4	37.1	29.1	45.4
Former smoker	45.8	49.8	36.2	48.0	45.5	49.8
Current smoker	24.6	43.1	47.4	49.9	25.3	43.5
Alcohol consumption frequency last year						
None	45.7	49.8	39.5	48.9	45.5	49.8
Once a year up to once a week	25.5	43.6	33.8	47.3	25.8	43.8
Twice a week up to daily^a^	28.5	45.1	26.6	44.2	28.4	45.1
Missing^a^	0.2	4.9	0.1	4.2	0.2	4.9
Socioeconomic						
Household income below Low Income Measure (LIM)	33.8	47.3	51.5	50.0	34.4	47.5
Indigenous status						
Non-Indigenous	96.2	19.2	94.5	22.8	96.1	19.3
Indigenous	3.2	17.7	4.9	21.6	3.3	17.9
Missing^a^	0.6	7.7	0.7	7.8	0.6	7.7
Homeownership						
Renter	27.5	44.7	48.8	50.0	28.2	45.0
Homeowner	72.3	44.8	51.4	50.0	71.6	45.1
Highest education in household						
High school incomplete	11.4	31.8	15.2	35.9	11.6	32.0
High school graduate^a^	15.7	36.3	15.8	36.4	15.7	36.3
Some college^a^	4.8	21.4	6.6	24.9	4.9	21.5
College degree	63.8	48.0	57.1	49.5	63.6	48.1
Missing^a^	4.2	20.2	5.3	22.6	4.3	20.2
Household type						
Couples with children	23.9	42.7	11.1	31.3	23.5	42.4
Couples without children	41.9	49.3	36.7	48.2	41.7	49.3
Single parents^a^	7.2	25.9	9.5	29.3	7.3	26.0
Individuals and other types	26.7	44.2	42.5	49.4	27.2	44.5
Missing^a^	0.3	5.1	0.5	6.9	0.3	5.2

Notes: All percentages were weighted by the individual sample weights. All differences between those who died before 65 and those who died at or after 65 were significant at *p* < 0.05 except the categories denoted by ^a^. According to Statistics Canada’s vetting rules, number of persons was rounded to the nearest digit of five while number of weighted population was rounded to the nearest hundred. The low proportions missing smoker status or homeownership were hidden to protect identity

**Fig. 2:**
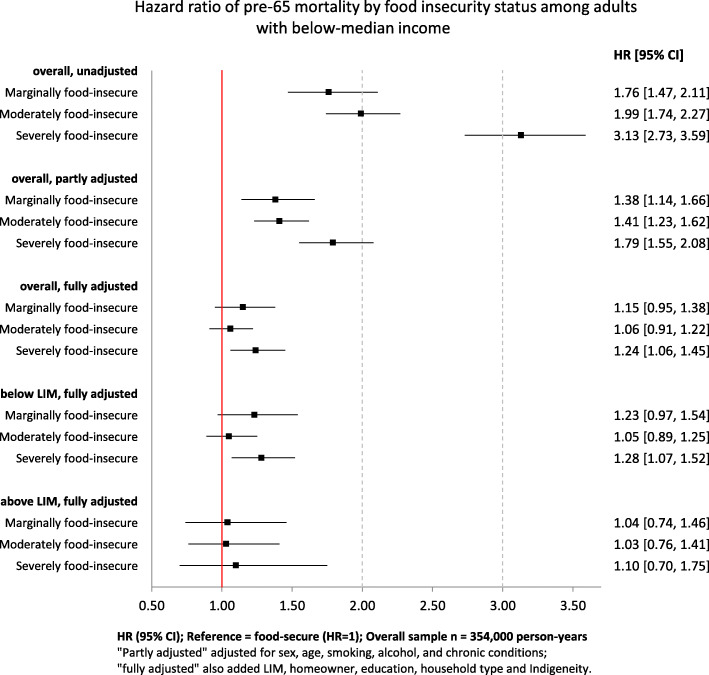
Hazard ratio of pre-65 mortality by food insecurity status among adults with below-median income. Hazard ratio of pre-65 mortality by food insecurity status was estimated using Cox proportional hazard model, with “food-secure” as the reference category (*N* = 354,000 person-years). Three sets of models were fitted on the full sample with below-median income, adjusting for no covariate, part of the covariates (respondent’s sex, age at interview, smoker status, alcohol consumption, number of chronic conditions), and all covariates (respondent’s sex, age at interview, smoker status, alcohol consumption, number of chronic conditions, Low Income Measure (LIM) status, homeownership, education attainment, household type, Indigenous identity). Fully adjusted models were further fitted on subsamples below and above LIM. Age at interview and number of chronic conditions were set as stratifying variables in all adjusted models due to their violation of hazard proportionality assumption indicated by Schoenfeld test

In the unadjusted model, marginal, moderate, and severe food insecurity were associated with 1.76, 1.99, and 3.13 times higher hazard of dying prior to 65, respectively, compared to food security (Fig. 2). The hazard ratios after adjustment for demographic and health-related factors were 1.38 (95% CI: 1.14, 1.66), 1.41 (95% CI: 1.23, 1.62), and 1.79 (95% CI: 1.55, 2.08) for marginal, moderate, and severe food insecurity, respectively. Severe food insecurity remained a significant predictor of pre-65 mortality after further adjusting for socioeconomic characteristics (HR: 1.24; 95% CI: 1.06, 1.45); marginal and moderate food insecurity did not show significance. Limiting the sample to below-LIM adults barely changed the results (HR: 1.28 for severe food insecurity; 95% CI: 1.07, 1.52). No significant association between food insecurity and mortality was found among above-LIM adults.

## Discussion

While prior studies have documented an association between moderate and severe food insecurity and mortality at all ages [25, 26], our analyses established the association between severe food insecurity and death before age 65 -among low-income adults approaching retirement age. Among low-income adults who died during follow-up, those from severely food-insecure households died on average 10 months earlier than their food-secure counterparts. No significant relationship was found for adults from households with income above LIM; nor was there any association between marginal or moderate food insecurity and mortality.

Low income is a strong predictor of food insecurity, yet the two factors capture substantially different aspects of economic hardship with limited overlap [34, 35]. Past research on food insecurity and mortality has not stratified the samples by income [24–26]. Our stratification yielded a significant association between food insecurity and pre-65 mortality among low-income adults. Considered in tandem with prior Canadian research documenting the protective effect of public pensions on food insecurity risk among low-income adults [5], our findings suggest that those whose food insecurity would most likely be alleviated by the receipt of public pensions are least likely to live long enough to collect the benefits. One explanation for our findings is the more pervasive and extreme material deprivation associated with low income. This was captured in our study through the measure of severe food insecurity, but it must also include housing instability and cost-related medication nonadherence [20, 36, 37], which could compound the effects of food insecurity and lead to health deterioration. For instance, poor housing conditions have been associated with respiratory hospitalizations among First Nation people in Canada [38]; precariously-housed Canadian youth were more likely to have substance addiction and mental disorders [39]; nearly one-quarter of the Canadian adults with difficulty affording prescription drugs reported use of health care services as a consequence [29]. That being said, the absence of a significant association among above-LIM adults may be due to the lack of statistical power given that food insecurity, especially its severe form, is relatively rare in households with incomes above LIM [35]. More research is needed to understand the co-occurrence of material hardships across income groups and their potential impacts on health.

Our findings also raise the question of the causal mechanism underpinning the elevated mortality observed among severely food-insecure adults of pre-retirement age. Seniors and non-seniors have been clustered together in past research linking food insecurity to vital status [24–26]. Our exploratory analyses found that severely food-insecure adults from our sample were more likely than their food-secure counterparts to die from infectious-parasitic disease, unintentional injuries and suicides although chronic conditions claimed the great majority of pre-65 deaths irrespective of one’s food insecurity status (results not shown due to limited death tolls). While beyond the scope of this study, these exploratory findings resonated with earlier research [24]. It will be important for future research to compare the causes of death associated with food insecurity by age groups and determine the causal pathways connecting food insecurity to premature mortality.

Our findings illustrate the seriousness of the health disadvantage faced by lower-income food-insecure adults approaching retirement age. While there is evidence suggesting that the public pension system alleviates food insecurity and improves overall health and mental health for low-income seniors over 65 [5, 12], those experiencing severe food insecurity are more likely than others to die before becoming eligible for the pension. Canada has seen food insecurity among low-income households reduced following policy interventions that have improved household financial resources [40–44]. However, many of these interventions are programs targeting households with children. For adults approaching retirement age with no children under 18, the only social programs available to mitigate material hardships caused by income shocks related to unemployment, illnesses, accidents, and other unpredictable events are Employment Insurance, workers’ compensation and social assistance. Yet, participation in these income assistance programs is associated with markedly elevated risks of food insecurity [2]. We urge the policymakers to reassess the coverage and adequacy of public assistance to working-age adults, so the most disadvantaged among them can make ends meet while transitioning into the retirement stage.

The use of a large population-representative sample, validated food insecurity measure, and administrative records of deaths are all strengths of our study. However, we also recognize limitations of our study. We had to exclude the jurisdiction-years that opted out of the food security module, which limited our statistical power and estimate precision. We also excluded adults 52 years old or younger, whose food insecurity status may be differentially associated with mortality compared to observations from this study. Moreover, our analysis does not establish causality: unobserved injuries and illnesses may have precipitated food insecurity and pre-65 death at the same time. Although we controlled for self-reported chronic conditions and lifestyle, these covariates were subject to recall and desirability biases and their measure was also cross-sectional. Incorporating clinical records may enhance the measurement precision in future studies. Also, we cannot account for the duration of food insecurity and effectively assumed that the baseline food insecurity status would persist to the end of follow-up. Longitudinal measurement of food insecurity will help ascertain causal relationships and differentiate chronic from acute food insecurity. Due to sample constraints, we did not stratify our sample by household structure despite the possible divergences in food-insecurity-mortality association among unattached adults, single mothers, and married couples [1, 2].

## Conclusions

Severe food insecurity was associated with elevated risk of deaths before 65 among low-income Canadians approaching retirement age. Our findings illustrate the serious health challenges faced by the most disadvantaged in the society, reinforcing food insecurity as a social determinant of health. Policymakers need to reassess the coverage and adequacy of income supports to the low-income working-age adults to ensure their food security and healthy ageing. Future research may differentiate the food-insecurity-mortality association by age and income levels and investigate the causal pathway between food insecurity and mortality.

## Supplementary information

**Additional file 1 **: **Supplementary table**. Jurisdictions that opted out of food security monitoring in the CCHS cycles 2005–15. Provinces and territories with no food insecurity measurement between the cycles 2005–06 and 2015 were denoted; these jurisdiction-cycles were categorically excluded from our analyses.

**Additional file 2 **: **Supplementary figure**. Sample selection process. A step-by-step illustration on the sampling of the CCHS respondents included in our analyses and the corresponding exclusion criteria.

## Data Availability

The data that support the findings of this study are accessible through Statistics Canada but restrictions apply to data access. For the current study, the data were accessed under contract through the Statistics Canada Research Data Centre at the University of Toronto.

## Abbreviations

CCHS: Canadian Community Health Survey
CVSD: Canadian Vital Statistics Database
CI: Confidence Interval
HR: Hazard Ratio
LIM: Low Income Measure
OAS: Old Age Security
GIS: Guaranteed Income Supplement
