# Supplementary material for: Severe food insecurity associated with mortality among lower-income Canadian adults approaching eligibility for public pensions: a population cohort study
Source: BMC Public Health. 2020 Oct 1;20:1484. doi: 10.1186/s12889-020-09547-y (PMC7528377; doi:10.1186/s12889-020-09547-y)
Supplement: Supplementary file 1 — Additional file 1 : Supplementary table. Jurisdictions that opted out of food security monitoring in the CCHS cycles 2005–15. Provinces and territories with no food insecurity measurement between the cycles 2005–06 and 2015 were denoted; these jurisdiction-cycles were categorically excluded from our analyses. [file 12889_2020_9547_MOESM1_ESM.docx]

| Supplementary Table. Jurisdictions that opted out of food security monitoring in the CCHS cycles 2005-15 | | | | | | | |
| --- | --- | --- | --- | --- | --- | --- | --- |
|  | 2005-06 cycle | 2007-08 cycle | 2009-10 cycle | 2011-12 cycle | 2013-14 cycle | 2015 cycle |  |
| Newfoundland and Labrador | X |  |  |  | X | X |  |
| Prince Edward Island |  |  | X |  |  |  |  |
| New Brunswick | X |  | X |  |  |  |  |
| Nova Scotia |  |  |  |  |  |  |  |
| Quebec |  |  |  |  |  |  |  |
| Ontario |  |  |  |  |  | X |  |
| Saskatchewan | X |  |  |  |  |  |  |
| Manitoba | X |  |  |  | X |  |  |
| Alberta |  |  |  |  |  |  |  |
| British Columbia |  |  |  |  | X |  |  |
| Yukon | X |  |  |  | X | X |  |
| Northwest Territories |  |  |  |  |  |  |  |
| Nunavut |  |  |  |  |  |  |  |
| Note: "X" denotes opt-out from food security monitoring in a given cycle. | | | | | | | |
