# Supplementary material for: Severe food insecurity associated with mortality among lower-income Canadian adults approaching eligibility for public pensions: a population cohort study
Source: BMC Public Health. 2020 Oct 1;20:1484. doi: 10.1186/s12889-020-09547-y (PMC7528377; doi:10.1186/s12889-020-09547-y)
Supplement: Supplementary file 2 — Additional file 2 : Supplementary figure. Sample selection process. A step-by-step illustration on the sampling of the CCHS respondents included in our analyses and the corresponding exclusion criteria. [file 12889_2020_9547_MOESM2_ESM.docx]

**Supplementary Figure.** Sample selection process

567,255 respondents from CCHS Sharelink 2005-15

Excluding:

63,830 respondents from jurisdiction-years with no food insecurity measurement

5,315 respondents with missing food insecurity level

120,475 seniors 65 years or older

266,215 individuals 51 years or younger

60,640 respondents with household income above national median (i.e. twice the LIM)

Merging in:

49,045 death records from CVSD 2005-17

Analyses:

354,000 person-years from 50,780 adults aged 52 to 64 at CCHS interview, 2,075 of whom died by 2017 before reaching 65.

567,255 respondents from CCHS 2005-15 merged with CVSD records
